# Persistent Organic Pollutant Residues in Human Fetal Liver and Placenta from Greater Montreal, Quebec: A Longitudinal Study from 1998 through 2006

**DOI:** 10.1289/ehp.0800205

**Published:** 2008-12-10

**Authors:** Josée Doucet, Brett Tague, Douglas L. Arnold, Gerard M. Cooke, Stephen Hayward, Cynthia G. Goodyer

**Affiliations:** 1 Health Canada, Health Products and Food Branch, Ottawa, Ontario, Canada;; 2 McGill University Health Centre–Montreal Children’s Hospital Research Institute, Montreal, Quebec, Canada

**Keywords:** fetus, human, liver, placenta, OCs, PBDEs, PCBs, POPs

## Abstract

**Background:**

There is general concern that persistent organic pollutants (POPs) found in the environment, wildlife, food, water, house dust, human tissues, and fluids may alter normal human physiologic activities (e.g., fetal development, immune and endocrine systems). Although the levels of some POPs [polychlorinated biphenyls (PCBs) and organochlorine pesticides (OCs)] in these matrices have decreased after their ban, others [polybrominated diphenyl ethers (PBDEs)] have increased in recent years.

**Objective:**

To determine the longitudinal trend of specific POPs in human fetal tissues for risk assessment purposes.

**Methods:**

We analyzed early to mid-gestation fetal liver (*n* = 52) and placental (*n* = 60) tissues, obtained after elective abortions during 1998–2006, for selected PBDEs, PCBs, and OCs using gas chromatography–mass spectroscopy.

**Results:**

Total PBDEs in fetal liver increased over time (mean ± SE: 1998, 284.4 ± 229.8 ng/g lipid; 2006, 1,607.7 ± 605.9; *p* < 0.03), whereas placental levels were generally lower, with no clear trend. Low levels of PCBs and OCs varied yearly, with no evident trend. The major analytes in 1998 were OCs (liver, 49%; placenta, 71%), whereas the major analytes in 2006 were PBDEs (liver, 89%; placenta, 98%). The 1998–2006 tissue PBDE congener profile is similar to that of DE-71, a commercial primarily pentabrominated diphenyl ether mixture manufactured in North America.

**Conclusions:**

Although commercial production of penta- and octa-brominated diphenyl ethers in North America was halted in 2004, their concentrations in fetal liver and placenta are now greater than the tissue burdens for the analyzed OCs and PCBs. Our findings also demonstrate that PBDEs accumulate within the fetal compartment at a very early stage in gestation.

Persistent organic pollutants (POPs), such as organochlorine pesticides (OCs) and polychlorinated biphenyls (PCBs), contaminate many environmental matrices and can be found in human blood, adipose tissue, and breast milk (Guvenius et al. 2003; Solomon and Weiss 2002). In addition, these POPs cross the placenta and have been reported to reduce gestational length and birth weight and to affect fetal development (Ando et al. 1985; Guvenius et al. 2003; Lanting et al. 1998; Rogan et al. 1986; Shen et al. 2007; Taylor et al. 1984). Furthermore, some of these chemicals may induce endocrine effects, and others are classified as “reasonably anticipated to be” or “known” human carcinogens (Carpenter 2006). Multiple studies have shown that the levels of several POPs found in the environment, human fat, maternal and cord bloods, and breast milk have decreased after being banned (Bates et al. 2002; Craan and Haines 1998; Norén and Meironyte 2000; Pereg et al. 2003; Schade and Heinzow 1998; Solomon and Weiss 2002). However, this is not universal; for example, concentrations of PCBs in Great Lakes fish have continued to oscillate (Bhavsar et al. 2007).

A more recently recognized class of environmental contaminant, whose chemical structure is similar to PCBs, is the brominated flame retardants, which includes the polybrominated diphenyl ether (PBDE) congeners (Gill et al. 2004; Hites 2004). For the past two to three decades, they have been added to plastics, polyurethane foam, textiles, and electronic equipment to prevent ignition or to slow the burn rate if a fire occurs. In animal studies, PBDEs have been found to affect neural and reproductive development as well as thyroid function (Birnbaum and Staskal 2004; Gill et al. 2004; Legler 2008; McDonald 2002; Talsness 2008). Although many of these investigations used pharmacologic doses of the PBDEs, more recent studies have demonstrated that exposure at doses relevant for humans at critical times during development can have effects on the endocrine and neural systems (reviewed in Legler 2008; Talsness 2008). Only two clinical epidemiologic studies have been published to date on the potential effects of *in utero* exposure and fetal outcome. Chao et al. (2007) recently reported that elevated levels of PBDEs in breast milk correlated significantly with lower newborn birth weight, birth length, chest circumference, and body mass index. In addition, Main et al. (2007) have found significantly higher PBDE levels in the breast milk of mothers whose newborn sons had cryptorchidism. One study of young adult males has reported a significant negative association between serum brominated diphenyl ether congener BDE-153 levels and testis size as well as sperm concentration (Akutsu et al. 2008).

In contrast to OCs and PCBs, many studies worldwide have reported that the amount of PBDEs found in the environment, human serum, and breast milk has been increasing steadily since the 1980s (Akutsu et al. 2003; de Wit et al. 2006; Li et al. 2006; Lorber 2008; Norén and Meironyte 2000; Pereg et al. 2003; Ryan et al. 2002; Schecter et al. 2004; Solomon and Weiss 2002). A few investigators in Europe have found that the PBDE levels in European samples peaked in 1997–1998 and subsequently have plateaued or declined, likely due to the earlier limitations on the production and use of penta- and octa-BDEs within the European Union versus North America (Darnerud et al. 2002; Law et al. 2006, 2008).

Human breast milk is only one source of environmental contaminants for a developing infant, and from a toxicology and regulatory perspective, it is the infant’s total body burden that is of greatest interest. Very little is known about when *in utero* exposure begins to contribute significantly to an infant’s body burden of PBDEs and other POPs. To examine this question, we analyzed the levels of multiple OCs *p*,*p*′-DDT (1,1,1-trichloro-2-[*o*-chlorophenyl]-2-2[*p*-chlorophenyl] ethane), *p*,*p*′-DDE (1,1-dichloro-2,2-bis[*p*-chlorophenyl] ethylene), *trans*-nonachlor, and hexachlorobenzene (HCB)], PCB congeners (49, 99, 118, 137, 138, 153, and 180), and BDE congeners (47, 85, 99, 100, 153, 154, and 183) in early to mid-gestation fetal liver and placental specimens obtained between 1998 and 2006. The results are an expanded version of data presented previously at two scientific meetings (Doucet et al. 2004, 2007).

## Materials and Methods

### Sample collection and storage

We obtained human fetal liver and placental (villous region) samples (fetal age, 9.5–20 weeks) after elective pregnancy terminations during 1998–2006 in the Greater Montreal area of Quebec with informed written consent. Fetal ages were determined by foot length (Munsick 1984). The tissues were placed in sterile vials and flash-frozen in a dry ice/acetone bath, initially stored at McGill University (−80°C), and then transferred in dry ice to Health Canada for processing. We collected a total of 52 liver and 60 placental samples, with 36 paired samples from the same fetuses. No information was available concerning the mothers because the tissues were collected anonymously. Ethics approval for the collection and use of the human fetal tissues was obtained from McGill University and Health Canada.

### Analyte/lipid extraction

We precleaned all glassware, except volumetric flasks and custom-made columns, in a dishwasher and baked them at 475°C for 2 hr in a Tempyrox oven (Tempyrox Co., Dallas, TX, USA). The volumetrics and custom-made columns were cleaned by solvent rinsing with acetone followed by hexane. We thawed approximately 1 g of each sample at room temperature in 50-mL centrifuge tubes and homogenized them in 20 mL of a 2:1 acetone/hexane solution for 1 min using a Polytron homogenizer (Brinkmann, Mississauga, ON, Canada). The resulting supernatant was quantitatively transferred and filtered through precleaned (dichloromethane) glass wool, collected in 250-mL round-bottom flasks (RBFs), and concentrated to approximately 1 mL on a rotary evaporator (Buchii, New Castle, DE, USA). The extracts were dried by pouring them into funnels containing prebaked sodium sulfate (700°C, overnight) and again collected in 250-mL RBFs. We then transferred the dried extracts to preweighed 10-mL vials for gravimetric lipid determinations.

### Sample cleanup

We performed sample cleanup on a 12-mm outer-diameter × 22-cm glass column containing 2% water-deactivated Florisil. Extracts were eluted using 70 mL hexane. This fraction contained BDE congeners (28, 47, 85, 99, 100, 153, 154, and 183), PCB congeners (49, 99, 118, 137, 138, 153, and 180), and several OCs (*p*,*p*′-DDT, *p*,*p*′-DDE, *trans*-nonachlor, HCB). We selected these analytes based on previous studies of Canadian breast milk samples showing that they had the highest levels and occurrences in 1992 and 2002 (Newsome et al. 1995; Ryan et al. 2002). In addition, the PBDE congeners matched those in the commercial mixture DE-71 (Chemtura Corp., Middlebury, CT, USA), which contains primarily penta-BDEs (Table 1). The Florisil extracts were transferred to 2-mL tapered chromatography tubes (Kontes, Vineland, NJ, USA) for concentration under vacuum. Liver extracts were concentrated to 300 μL and the placental extracts to 100 μL for gas chromatograph–mass spectrometer (GC-MS) injection.

### Sample analysis

We analyzed samples using an Agilent 6890N/5973 GC-MS system operated in electron impact ionization mode equipped with an Agilent 7683 autosampler (Agilent Technologies, Mississauga, ON, Canada). Aliquots of 2 μl were injected via an on-column injector operated in oven-track mode. The capillary column employed was a 30 m × 0.25 mm × 250 μm DB5-MS (Agilent Technologies) coupled to a 2-m × 0.53-mm deactivated fused silica column (Chromatographic Specialties, Brockville, ON, Canada). The GC oven parameters were 80°C for 2 min; 8°C/min to 220°C, 4 min hold; 5°C/min to 230°C; 10°C/min to 295°C; 10 min hold. The helium carrier gas was set at a constant flow rate of 1.2 mL/min and the transfer line temperature was maintained at 295°C (MS source, 250°C; MS quad, 150°C). Supplemental Material, Table 1 (http://www.ehponline.org/members/2008/0800205/suppl.pdf) provides the mass-to-charge ratios monitored for the target ions and qualifier ions as well as the expected qualifier response factors and response factor tolerances.

### Calibration and quality control

External standard calibration was used after determination of the instrument’s linear response range for each analyte. We based positive identification of target compounds on three criteria: a chromatographic retention time matched to analytical standards, a signal-to-noise ratio of > 3, and qualifier ion ratios within the tolerances listed in Supplemental Material, Table 1 (http://www.ehponline.org/members/2008/0800205/suppl.pdf). Analyte concentrations in the samples were calculated based on the equation of the linear regression line after forcing through the origin. The response was linear for all analytes (*r*^2^ > 0.99).

Instrument sequences consisted of one reagent blank, one corn oil sample (no background POPs) spiked with the analytes, one standard reference material (SRM) sample (WMF-01 freeze-dried fish tissue; Wellington Laboratories, Guelph, ON, Canada), and seven tissue samples [see Supplemental Material, Figures 1–5 (http://www.ehponline.org/members/2008/0800205/suppl.pdf)]. The BDE background contamination levels fluctuate almost daily, as determined from the reagent blanks; therefore, we subtracted this value from the spike, the SRM, and the tissue samples on a batch-to-batch basis. Control charts were used to monitor the performance of the SRM and corrective action was undertaken any time the warning limit (mean ± 2σ) was approached. Recoveries from the spiked corn oil sample were also monitored on a batch-to-batch basis. Supplemental Material, Table 2, provides mean recovery values (http://www.ehponline.org/members/2008/0800205/suppl.pdf). Method detection limits (MDLs) for each analyte were determined using eight corn oil samples (100 mg) spiked with a known concentration of each analyte; linearity was established between 7 and 900 ng/mL [Supplemental Material, Figure 3 (http://www.ehponline.org/members/2008/0800205/suppl.pdf)]. The corn oil spikes were then extracted as outlined above and the mean concentrations for each analyte calculated. We calculated the MDL as the SD for the replicates (*n* = 8) multiplied by 2.31 (Student’s *t*-value for 8 degrees of freedom). Supplemental Material, Table 2, lists MDLs (http://www.ehponline.org/members/2008/0800205/suppl.pdf).

This extraction methodology has been used in our laboratory for many years to evaluate POPs in various matrices (Bondy et al. 2003, 2004). The GC-MS method allows for the concurrent analysis and quantification of several OC pesticides and PCB and PBDE congeners, facilitating extensive data acquisition from small specimens.

### Statistical analysis

**Figure 1:**
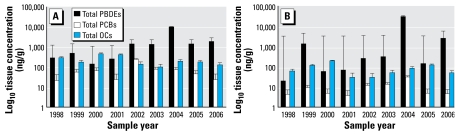
Yearly levels of total PBDE congeners (47, 85, 99, 100, 153, 154, and 183), total PCB congeners (49, 99, 118, 137, 138, 153, and 180), and total OCs (DDT, DDE, *trans*-nonachlor, HCB) in human fetal liver (*A; n* = 4–8/year) and human fetal placenta (*B; n* = 4–10/year) obtained from elective terminations of 9.5- to 20-week fetuses in the Greater Montreal area of Quebec during 1998–2006. The data are mean ± SE (log_10_ concentrations, ng/g lw) for all of the samples analyzed.

**Figure 2:**
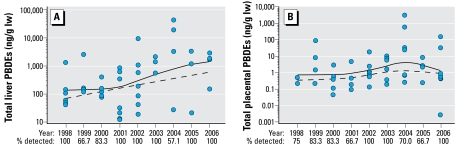
Log_10_ concentrations of total PBDEs (ng/g lw) in human fetal liver (*A*) and placenta (*B*) from 1998 through 2006. The scatter plots illustrate the samples with detectable PBDEs. Numbers below the *x*-axis are the percentages of samples per year that had detectable levels. The dashed line represents the trend over the 9-year period using all of the samples per year (i.e., including zeros), and the solid line shows the trend using only samples with detectable levels of PBDEs. Statistical analyses of trends were done using the data from all samples.

**Figure 3:**
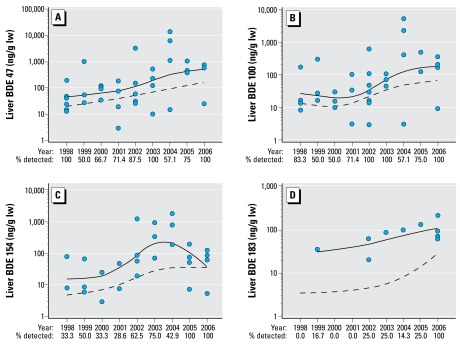
Log_10_ concentrations (ng/g lw) of BDE-47 (*A*), BDE-100 (*B*), BDE-154 (*C*), and BDE-183 (*D*) in human fetal liver during 1998 through 2006. The scatter plots illustrate the samples with detectable congeners. Numbers below the *x*-axis are the percentages of samples per year that had detectable levels. The dashed line represents the trend over the 9-year period using all of the samples per year (i.e., including zeros), and the solid line shows the trend using only liver with detectable levels of each BDE congener. Statistical analyses of trends were done using data from all samples.

**Figure 4:**
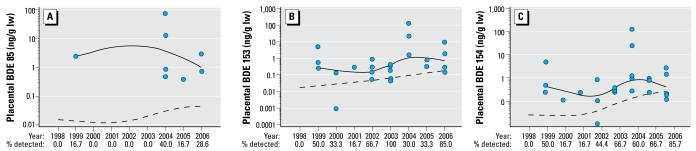
Log_10_ concentrations of BDE congeners (ng/g lw); BDE-85 (*A*), BDE-153 (*B*), and BDE-154 (*C*) in human placenta from 1998 through 2006. The scatter plots illustrate the samples with detectable congeners. Numbers below the *x*-axis are the percentages of samples per year that had detectable levels. The dashed line represents the trend over the 9-year period using all of the samples per year (i.e., including zeros), and the solid line shows the trend using only placenta with detectable levels of each BDE congener. Statistical analyses of trends were done using data from all samples.

The data in Figure 1 are log_10_ concentrations [nanograms per gram lipid weight (lw); mean ± SE] of total PBDEs, PCBs, and OCs. Figures 2–4 present the data on total and individual PBDE congeners as scatter plots (log_10_ tissue concentration, nanograms per gram lipid); the dashed line represents the trend with time (1998–2006) using all of the samples per year (i.e., including zeros), and the solid line, the trend using only samples with detectable levels of PBDEs. We analyzed the data using a nonparametric approach because of the existence of zeros (nondetects) and values below the MDL that prevented a transformation from achieving normality. The nonparametric measure of correlation, Kendall’s tau statistic (Daniel 1978), was used to test for trends. When using a nonparametric approach, the use of a zero or a value below the MDL does not affect the results. The SAS version 9.1 program (SAS Institute Inc., Cary, NC, USA) was used to create the trend curves in Figures 2–4.

## Results

**Figure 5:**
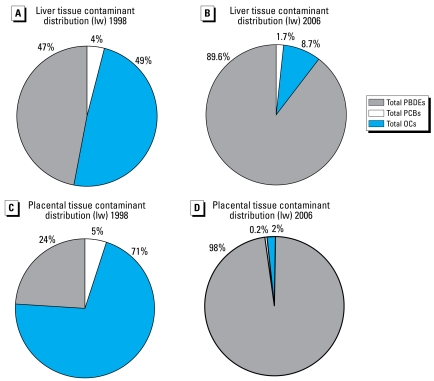
Comparison of the percentage distribution for total PBDE congeners (47, 85, 99, 100, 153, 154, and 183), total PCB congeners (49, 99, 118, 137, 138, 153, and 180), and total OCs (DDT, DDE, *trans*-nonachlor, HCB) in human fetal liver and placenta obtained from elective terminations of 11.25- to 20-week fetuses in the Greater Montreal area of Quebec during 1998 [(*A*) liver, *n* = 6; (*C*) placenta, *n* = 4; 13.8- to 20-week fetal age] and during 2006 [(*B*) liver, *n* = 4; (*D*) placenta, *n* = 7; 11.25- to 16.5-week fetal age], for all the samples analyzed.

Figure 1 shows the total amounts of PBDEs, PCBs, and OCs on a lipid basis in our fetal liver and placental samples from 1998 through 2006. In general, the levels of these POPs were severalfold higher in the liver than the placenta. The marked “within-year” variations occurred because some specimens had little or no detectable analyte, whereas others from the same year contained large amounts (Figures 2–4). This was especially noticeable with PBDEs in the placenta, where one sample might have undetectable levels of PBDEs, and another, two to three log units (Figures 2 and 4). However, even with this variability, the average relative concentration for each of the BDE congeners from 1998 through 2006 was very similar to the commercial mixture DE-71, which contains primarily pentabrominated diphenyl ethers, with congeners BDE-99, BDE-47, and BDE-100 as the major contributors (Table 1). In addition, we found marked changes during the tissue collection period regarding the relative amounts of OCs, PCBs, and PBDEs in the liver and placenta: the major contaminants were OCs in 1998 but PBDEs in 2006 (Figure 5).

Examination of the “zero” samples showed that all tissue samples had detectable PBDE, PCB, and/or OC contaminants and that more placental samples than liver samples showed undetectable PBDEs, PCBs, or OCs (Figure 2). We found no significant trends for the number of liver or placental samples with undetectable PBDEs, PCBs, or OCs (Figures 2–4); only 2 of the 36 paired liver and placental samples showed a parallel lack of PBDEs, and only one paired sample showed a parallel lack of PCBs (data not shown).

Kendall’s tau test for trend revealed no significant changes from 1998 through 2006 in any of the individual OC analytes or total OCs in either the fetal liver or placental tissues. The major OC in both tissues was *p*,*p*′-DDE [see Supplemental Material, Table 3 (http://www.ehponline.org/members/2008/0800205/suppl.pdf)]. Of the PCBs, only PCB 180 showed a significant trend: a decrease with time and only in liver (*r* = −0.35, *p* < 0.003). The major PCB congener in both tissues was PCB 153 [see Supplemental Material, Table 3 (http://www.ehponline.org/members/2008/0800205/suppl.pdf)].

In contrast, the total PBDE concentration significantly increased in liver during the sampling period (*r* = 0.22, *p* < 0.03; Figure 2A), as did several of the individual congeners: BDE-47 (*r* = 0.20, *p* < 0.04; Figure 3A), BDE-100 (*r* = 0.24, *p* < 0.02; Figure 3B), BDE-153 (*r* = 0.29, *p* < 0.006; data not shown but similar to BDE-154), BDE-154 (*r* = 0.29, *p* < 0.007; Figure 3C), and BDE-183 (*r* = 0.35, *p* < 0.002; Figure 3D). The increase in BDE-99 was not quite significant (*r* = 0.19, *p* < 0.065; data not shown). In the placental specimens, there was a significant increase in BDE-85 (*r* = 0.23, *p* < 0.04; Figure 4A), BDE-153 (*r* = 0.22, *p* < 0.03; Figure 4B), and BDE-154 (*r* = 0.31, *p* < 0.002; Figure 4C), but not total PBDEs (Figure 2B).

Kendall’s tau test for correlation found no correlations for any of the analytes in the 36 paired human fetal liver and placental samples (fetal age, 9.5–19 weeks). Because of the limited number of samples per year, it was not possible to analyze statistically whether there was a correlation between the age of the fetus and the amount of POPs in the tissue samples; however, we found no apparent associations when we graphed the data (data not shown).

## Discussion

The PBDE levels in human tissue and fluid specimens from North America are some of the highest in the world (Lorber 2008; Mazdai et al. 2003; Ryan et al. 2002; Schecter et al. 2004). This raises health and regulatory concerns because of the chemical similarities between PCBs and PBDEs, and because levels of PBDEs in human samples from North America appear to be rising, whereas they are generally static or currently decreasing in many other parts of the world (Birnbaum and Staskal 2004; Law et al. 2008; Lorber 2008). Of particular concern are the reports that young infants have higher tissue concentrations than do older children and adults, because it is generally believed that young infants are more vulnerable to toxicologic insults (Birnbaum and Hubal 2006; Fischer et al. 2006; Thomsen et al. 2002; Toms et al. 2008). *In utero* exposure is also considered to be a critical period (Chao et al. 2007; Main et al. 2007); however, there is a general scarcity of data regarding POP levels in fetal tissues. This hampers regulatory assessment of health risks during the earliest stages of human development.

Although several studies have analyzed the OC and PCB contents of fetal, newborn, and infant specimens (Carpenter 2006; Guvenius et al. 2003; Solomon and Weiss 2002), only a few groups have examined PBDEs, and they have focused primarily on the term newborn. Four studies in Europe and Asia have found that the levels of PBDEs in paired term cord and maternal sera are relatively low and quite similar (Antignac et al. 2008; Bi et al. 2006; Guvenius et al. 2003; Hirai et al. 2004). They reported median values for total tri- and tetra-BDEs ranging from 0.4 to 4.4 ng/g (lipid) in cord and maternal sera collected from 2000 to 2004 in Sweden, France, Japan, and China. Mazdai et al. (2003) found approximately 10-fold higher levels in the U.S. samples collected in 2001, but the levels in the maternal and cord sera were again similar, with a median of approximately 38 ng/g lipid. Three groups have analyzed term placental samples; they reported total PBDEs of approximately 1 ng/g (lipid) for tissues collected in Finland, Japan, and Denmark between 1994 and 2002 (Hirai et al. 2004; Main et al. 2007; Strandman et al. 2000). These studies provide insight into the transfer of PBDEs within the human feto-placental unit, suggesting that, at the end of gestation, there is a relatively free passage of PBDEs from the maternal blood across the placenta.

Only a North American study has examined PBDE levels in a nonplacental fetal tissue: Schecter et al. (2007) analyzed liver samples from 11 fetuses (19–37 week fetal age) in 2004 who were stillborn or died after premature delivery due to multiple congenital abnormalities. They found total PBDE median levels of 15.2 ng/g (lipid), with a range from 4.0 to 98.5 ng/g; there was no correlation between the hepatic PBDE levels and increasing fetal age. These PBDE levels were generally lower than what Schecter et al. (2004) had previously measured in non-pregnant adult female blood or breast milk from 2003 samples, or what Mazdai et al. (2003) found in North American maternal and cord samples from 2001. However, they were higher than serum or placental levels in European or Asian samples, suggesting that both fetal and maternal PBDE body burdens are higher in North America.

The fetal liver study by Schecter et al. (2007) was a cross-sectional investigation, looking only at samples obtained in 2004, and was limited to an analysis of PBDE congeners. To the best of our knowledge, the present study is the first to analyze fetal tissues for several POPs over a period of several years during which PBDE congener levels in human specimens have been rising exponentially in North America. Consequently, these data should be of benefit to agencies concerned with *in utero* exposure and health risks from these environmental contaminants.

The major findings from our study are as follows:

- Although there was little change in the concentrations of OCs or PCB congeners in fetal liver or placenta from 1998 through 2006, there was a significant increase in liver, but not in placenta, of PBDE congeners.
- In general, the levels of all of the contaminants were severalfold higher in the liver than in the placental tissues, and there was no correlation for any of the contaminants in paired samples.
- There was high variability in tissue contaminant levels, especially with the placental samples.

Our finding of a significant shift in Canadian fetal tissues from primarily OC/PCB contaminants to PBDEs in the period from 1998 through 2006 is not surprising. The widespread banning of several POPs that are environmental contaminants (e.g., DDT, PCBs) in the 1970s and 1980s has produced a decline in their relative concentrations in various matrices, including human specimens, during the past 30 years (Norén and Meironyte 2000; Pereg et al. 2003). In addition, although a limitation on the production and use of penta- and octa-BDEs in many countries in Europe beginning in the early 1990s has resulted in static or decreasing PBDE levels in European nonpregnant, maternal, and cord sera and in breast milk samples (Darnerud et al. 2002; Law et al. 2006, 2008), the manufacture and sale of these PBDE mixtures was halted in North America (by consent of the manufacturer) on 1 January 2004.

Since the 1990s, studies of environmental and human specimens in North America have uniformly documented a dramatic increase in PBDE levels (de Wit et al. 2006; Li et al. 2006; Pereg et al. 2003; Ryan et al. 2002; Schecter et al. 2004; Solomon and Weiss 2002). What is worrisome, based on our findings and those of others, is that these high levels appear to be passing freely from the mother to the fetus from very early in gestation, as well as postnatally through breast-feeding (Mazdai et al. 2003; Ryan and van Oostdam 2004; Schecter et al. 2007). For the past 20–30 years, PBDEs have been used as additives in many plastics, foams, and other long-lived products that have widespread use commercially as well as in the home. Despite the present lack of penta-and octa-BDE manufacturing in North America, these PBDEs will continue to be slowly released into the environment, concurrently contaminating the food chain, in the coming years. Personal lifestyle choices (e.g., food options), age, occupation, metabolism, and tissue burden before 2004 will all affect blood, breast milk, and tissue contaminant levels (Allen et al. 2007; Birnbaum and Hubal 2006; Birnbaum and Staskal 2004; Lorber 2008; McDonald 2002). It is encouraging, however, that although initial studies by Ryan et al. (2002) found that median levels of PBDEs in human milk samples collected across Canada had increased an order of magnitude from 1992 through 2002 (from 2.9 to 25.0 ng/g lipid), a more recent study analyzing breast milk samples from Hamilton, Ontario, found that the levels in 2005 samples were slightly, but not statistically, lower than the samples collected in Hamilton previously, suggesting that the amount of PBDEs in breast milk samples from that region of Canada may be starting to plateau (Ryan et al. 2006).

On average, the concentrations of PBDEs as well as OCs and PCBs in our liver samples were appreciably higher than those in the placenta, suggesting tissue-specific bioaccumulation. This may be due in part to the relative difference in their lipid content (our average for liver was 2.56%, versus 1.31% for placenta). However, the lack of a correlation within the paired samples in our study, as well as the results from investigations of paired maternal and cord sera (Antignac et al. 2008; Guvenius et al. 2003; Hirai et al. 2004; Mazdai et al. 2003), suggest that these lipophilic contaminants are passing relatively freely through the placental tissue and into the fetal circulation, regardless of their level of bromination, and then are likely accumulating in the liver over time. This concept is supported by the fact that the percentages of the seven BDE congeners we measured in both the placental and liver tissues closely resemble those found in the commercial DE-71 mixture. Thus, the placenta does not appear to provide much of a barrier for these contaminants at early to mid-gestation (present data) or at term (Antignac et al. 2008; Guvenius et al. 2003; Hirai et al. 2004; Mazdai et al. 2003).

Although 84% of our PBDE values were in the range of 0–1,500 ng/g lipid, two liver and three placental samples had levels approximately 10- to 100-fold higher. Unfortunately, these were not among the paired samples, but that may not have provided much insight into whether the mother’s BDE burden greatly exceeded the population’s average because we found no association between the amount of PBDEs in our fetal liver and placental samples. We analyzed samples collected anonymously, therefore neither our present study nor that of Schecter et al. (2007) has any information about maternal PBDE exposure that could help to explain the high levels.

Interestingly, ours is not the only report of PBDE concentrations that vary markedly among samples. In a review of Canadian breast milk studies, Gill et al. (2004) noted that PBDE concentrations differed by three orders of magnitude, a variability that they did not see with PCBs and dioxins. Both Gill et al. (2004) and Birnbaum and Hubal (2006) also observed that PBDE biomonitoring studies from several countries found “outliers” in diverse human specimens. More recently, researchers at the U.S. Centers for Disease Control and Prevention have determined PBDE concentrations in the blood of > 2,000 Americans: the highest levels were in the youngest group (12–19 year olds) (Sjodin et al. 2008). They also reported that the highest concentration in one subject’s blood (3,680 ng/g lipid) was more than 12 times the average value for the 95th percentile (291 ng/g lipid) and that 5% of their population had PBDE levels more than seven times the geometric mean. Although an explanation for such fluctuations was not readily apparent, factors such as proximity to the source of contamination, length of exposure, exposure to different PBDE commercial mixtures, and differences in individual nutritional status, absorption, metabolism, and excretion are likely contributing factors (Athanasiadou et al. 2008; Stapleton et al. 2008; Van den Steen et al. 2008; Zhao et al. 2008). Interestingly, the major source of PBDE exposure for most humans appears to be the dust associated with household and commercial consumer products (Athanasiadou et al. 2008; Lorber 2008).

In summary, our study of POPs in human fetal liver and placental tissues suggests that PBDEs, PCBs, and OCs are passing into and accumulating within the fetal compartment from a very early stage in gestation. Although we found little change in the relative amounts of the PCBs and OCs from 1998 through 2006, there has been a significant increase in the fetal liver levels of PBDEs. These data, along with the results from previous studies of human postnatal blood, breast milk, and adipose tissues, suggest increased exposure of humans to PBDEs at every stage in life during the past decade. Although this appears to be more of an issue in North America, the widespread presence of PBDEs in commercial and household items as well as in the environment and food chain ensures that this is a universal problem. Whether some individuals are reaching an exposure level that may constitute a danger to their health is unknown. Annual monitoring programs (e.g., blood, breast milk, fetal tissues, meconium, hair) in different regions would help to answer how the body burdens are changing in the future and provide important dose information for studies of the physiologic effects of these contaminants.

## Figures and Tables

**Table 1:** Comparison of the congener composition of DE-71 (commercially available PBDE mixture) and PBDEs found in human fetal liver and placenta from 1998 through 2006.

		Human fetal tissues (average for 1998–2006)	
BDE congener	DE-71^a^ (%)	Liver (%)	Placenta (%)
28	0.21	ND	ND
47	42–46	30.4	36.1
85	1.7	1.8	2.1
99	40–41	45.1	42.8
100	7.1–7.7	11.9	11.0
153	2.7–4.4	3.9	4.0
154	2.3–3.1	6.1	3.8
183	ND	0.8	0.3

ND, not determined.

a Based on analyses by the authors of DE-71 supplied by the Great Lakes Chemical Corporation.
